# Objectively measured levels of moderate to vigorous intensity physical activity are associated with cognitive impairment in diabetic hemodialysis patients: a cross-sectional study

**DOI:** 10.3389/fmed.2024.1466574

**Published:** 2024-10-16

**Authors:** Zhixin Zhang, Xiaoyu Chen, Siqin Gaowa, Ruiting Liang, Zhetong Jin, Ziyi Shi, Peipei Han, Cheng Lin, Xiaoli Wen, Peng Lin, Qi Guo

**Affiliations:** ^1^Department of Rehabilitation Medicine, Shanghai University of Medicine and Health Sciences Affiliated Zhoupu Hospital, Shanghai, China; ^2^Tianjin Key Laboratory of Exercise Physiology and Sports Medicine, School of Sports and Health, Tianjin University of Sport, Tianjin, China; ^3^Department of Rehabilitation Medicine, Shanghai University of Medicine and Health Sciences, Shanghai, China; ^4^Department of Cardiovascular, Inner Mongolia People's Hospital, Inner Mongolia, China; ^5^Department of Rehabilitation Medicine, School of Health, Fujian Medical University, Fujian Province, China

**Keywords:** physical activity, hemodialysis, accelerometer, diabetes, cognitive impairment

## Abstract

**Objective:**

The purpose of this study was to observe the relationship between objectively measured levels of physical activity and cognitive impairment (CI) in the presence or absence of diabetes in middle-aged and elderly hemodialysis patients.

**Methods:**

In this multicenter cross-sectional study, 339 clinically stable hemodialysis patients (210 males; mean age: 67.38 ± 8.07 years) aged ≥55 years were included from 7 dialysis units in Shanghai, China. The Chinese version of the Modified Mini-Mental State Examination (MMSE) was used to assess the CI. The duration of physical activity at different intensities, including moderate to vigorous physical activity (MVPA) as well as light physical activity (LPA), was measured using a triaxial accelerometer (ActiGraph GT3X+, Pensacola, FL, USA). Logistic regression and multiple linear regression were used for analyses.

**Results:**

The prevalence of CI was higher in hemodialysis patients with comorbid diabetes (24.3%). In diabetic patients, MVPA (increase per 10 min/day) was negatively associated with CI after adjusting for covariates [(OR = 0.89, 95%CI = 0.79–0.99), *p* = 0.042]. However, no significant association between physical activity and CI was found in non-diabetic hemodialysis patients. Further analyses revealed that MVPA was positively associated with temporal orientation, attention and calculation and recall in diabetic hemodialysis patients.

**Conclusion:**

Physical activity was associated with CI in diabetic hemodialysis patients rather than the non-diabetes group. This study is important for early differential diagnosis of CI and improvement of cognitive status in hemodialysis patients.

## 1 Introduction

Cognitive impairment (CI) is prevalent in the maintenance hemodialysis (MHD) population, with the prevalence of varying degrees of cognitive impairment ranging from 49.1 to 80.91%, which is more than three times higher than that of the population with normal renal function (1), and is attributed to the combination of its higher prevalence of cardiovascular (CV) risk factors and factors associated with renal disease (2). There is increasing evidence that combined cognitive impairment not only reduces hemodialysis patients’ own independence and adherence to dialysis and medication (3), but is also an independent risk factor for death in hemodialysis patients (4). Therefore, early recognition of cognitive impairment and assessment of modifiable factors associated with it is essential.

Physical activity (PA) is defined as bodily movements produced by skeletal muscles that result in energy expenditure (5). It has been reported that hemodialysis patients exhibit lower levels of physical activity (6) and are associated with an increased risk of death (7) compared to age-matched controls. Multiple studies have indicated that higher levels of physical activity are associated with beneficial maintenance of cognitive function in old age (8, 9). A recent report noted that time spent in moderate to vigorous physical activity (MVPA) was positively associated with cognitive areas such as executive ability and memory in middle-aged and older adults (9); however, other studies have not found an association between physical activity and reduced risk of cognitive impairment (10). Preliminary evidence now suggests an association between lower levels of physical activity and poorer cognitive function in hemodialysis patients (11, 12), but these studies have largely relied on self-report questionnaires to define physical activity. As a result, triaxial accelerometers, which can overcome the limitations of self-reporting and provide objective results for sedentary and varying-intensity physical activity times, have been widely used in research; however, it is worth noting that there is no precedent for using accelerometers to measure varying-intensity levels of physical activity in maintenance hemodialysis patients in our country, and even less in-depth investigation in relation to the disease.

In addition to physical activity, diabetes may also have a negative impact on cognitive function and is considered a major cause of end-stage renal disease (13). Recent studies have shown a significant association between longer periods of physical activity and a reduced risk of diabetes (14) and slower progression of diabetes to chronic kidney disease (CKD) (15). In addition, previous studies have shown that diabetes is associated with an increased risk of cognitive impairment in hemodialysis patients (16), which may be due to endothelial and cerebral microvascular dysfunction caused by hyperglycemia, thus affecting cognitive function (17); but there is also study that have not found a correlation between the two (18). Furthermore, it has also been shown that people with diabetes who have lower levels of physical activity face a higher risk of dementia compared to non-diabetics with higher levels of physical activity (19).Therefore, it is reasonable to assume that diabetes and low levels of physical activity may interact in some way to jointly impact on cognitive function. However, to date, no study has explored the relationship between objectively measured levels of physical activity and cognitive impairment in a hemodialysis population stratified by the presence or absence of diabetes, and therefore, the impact of the presence of diabetes on this relationship is unknown.

Therefore, the aim of this study was to investigate the relationship between objectively measured levels of physical activity of different intensities and cognitive dysfunction in middle-aged and elderly hemodialysis patients with or without diabetes. In addition, this study investigated the relationship between physical activity levels and specific cognitive functions in the presence or absence of diabetes to provide evidence for clinicians to effectively manage cognitive dysfunction in hemodialysis patients.

## 2 Materials and methods

### 2.1 Study subjects

This was a multicenter cross-sectional study that included patients undergoing hemodialysis from July 2020 to April 2021 at seven hemodialysis centers in Shanghai. Patients were older than 55 years of age, had been receiving maintenance hemodialysis for at least 3 months, and were able to provide informed consent. Patient exclusion criteria were as follows: (1) patients who refused to wear accelerometers or with missing data; (2) did not have a blood sample collected; (3) inability to communicate with the researchers or were unable to provide informed consent; and (4) had a known diagnosis of dementia, psychiatric illness, or other degenerative disease. After the exclusion of 36 subjects, the final analytic sample was 339 (211 males and 128 females). Twenty nine patients with missing or invalid watch data; 7 had missing questionnaires or no Mini-Mental State Examination (MMSE) scores. The study was approved by the Ethics Committee of Shanghai University of Medicine and Health Sciences and the methods were carried out in accordance with the principles of the Declaration of Helsinki. The informed consent was obtained from all patients prior to enrollment in the study.

### 2.2 Assessment of cognitive function

Cognitive function was evaluated by using the MMSE in this study, which was validated for Chinese seniors. It includes 30 items, and the score ranges from 0 to 30 points, with higher scores indicating better cognitive performance. The MMSE includes a broad set of cognitive domains that measure the following: orientation to time (five points), orientation to place (five points), registration (three points), attention and calculation (five points), recall (three points), and language (nine points) (20). Considering the significant correlation between education level and MMSE scores, in China, the cut-off points for defining cognitive impairment are 17/18, 20/21, and 24/25 for illiterate people, people with primary education, and people with education above middle school, respectively (21).

### 2.3 Physical activity and daily steps

Physical activity time and daily steps was measured using a wrist-worn triaxial accelerometer (ActiGraph GT3X+, Pensacola, FL, USA). Patients were asked to wear it for seven consecutive days, except for bathing and swimming moments (22). Accelerometer data were screened and analyzed using ActiLife software (version 6.0, Pensacola, FL, USA) with a sampling period of 60 s and a sampling frequency of 60 Hz, which was utilized to remove sleep time based on the subject’s sleep log. No accelerometer signal for more than 60 consecutive minutes was defined as “not worn,” accelerometer wear for at least 10 h per day was considered a valid wear day, and participants with at least four valid wear days in a week (including at least two full dialysis days and two non-dialysis days) were included in the analysis. Different levels of physical activity were categorized according to thresholds determined by Troiano et al. (23) where 100–2019 count/min was light physical activity (LPA) and ≥2020 count/min was MVPA.

### 2.4 Diabetes mellitus assessment

Access to diabetes information was based on subjects’ self-reports, and we again carefully checked the fasting plasma glucose (FPG) data through electronic medical records. According to the American Diabetes Association 2021 criteria, FPG level ≥ 7.0 mmol/L or 2-h plasma glucose ≥11.1 mmol/L during an oral glucose tolerance test or HbA1c ≥6.5% was considered as diabetes (24).

### 2.5 Covariates

All subjects were invited to participate in a face-to-face interview to answer a standardized questionnaire. Baseline socio-demographic characteristics, health behavior, and chronic disease prevalence were used as covariates. Demographic characteristics included age, gender, post-dialysis weight, age on dialysis, education level, and marital status. Health behaviors include smoking, drinking habits and physical function (TUGT). Comorbidity was assessed using the Charlson Comorbidity Index (CCI), which is a measure that explains multiple comorbidities by creating a summed score based on the presence of 19 comorbidities (25). We collected biochemical data including serum albumin, hemoglobin, calcium, phosphate and parathyroid hormone (PTH) within 3 months of the physical assessment. Dialysis adequacy was defined as total urea fraction clearance index (Kt/V).

### 2.6 Statistical analyses

Baseline characteristics of participants are presented according to the classification of diabetes and CI. Normally distributed continuous variables are presented as mean ± standard deviation (mean ± SD), and non-normally distributed data are presented as median (median), with interquartile spacing of 25 to 75% in parentheses. Categorical variables are expressed as numbers and percentages. Baseline socio-demographic characteristics were analyzed using t-test, Pearson’s chi-square test and Mann–Whitney U-test. Binary logistic regression was used to analyses the relationship between physical activity and CI in hemodialysis patients in the non-diabetic and diabetic groups. CI was used as the dependent variable, time spent in physical activity of different intensities (LPA, MVPA) as the independent variable, and adjustment for several confounders [age, gender, body mass index (BMI), education, widowhood, CCI and Kt/V] as covariates. Linear regression models were used to analyses the relationship between MVPA and each cognitive function. All statistical analyses were performed using SPSS V26.0 software, and differences were considered statistically significant at *p* < 0.05.

## 3 Results

**Figure 1:**
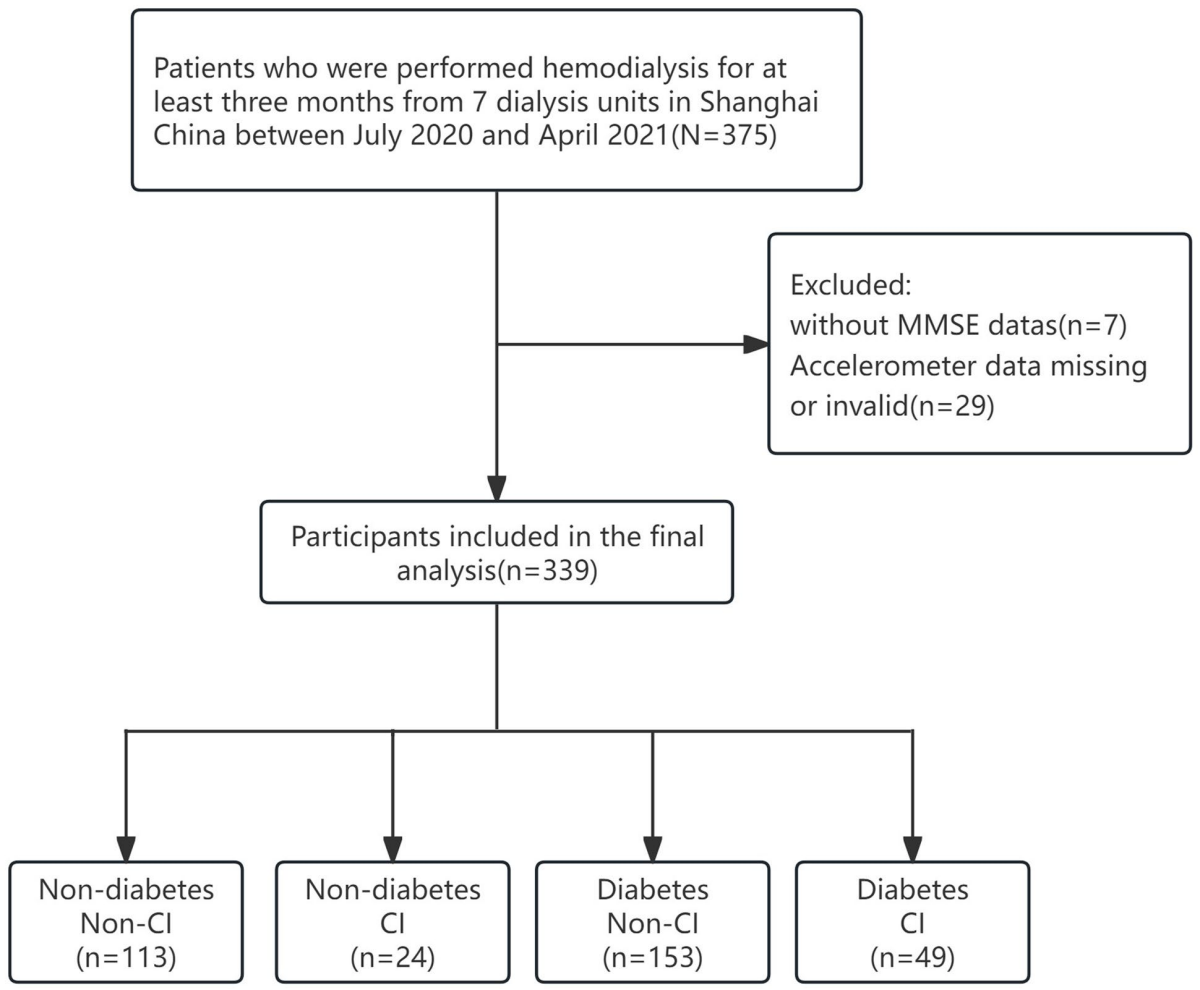
Flowchart of the study. CI, Cognitive Impairments.

**Figure 2:**
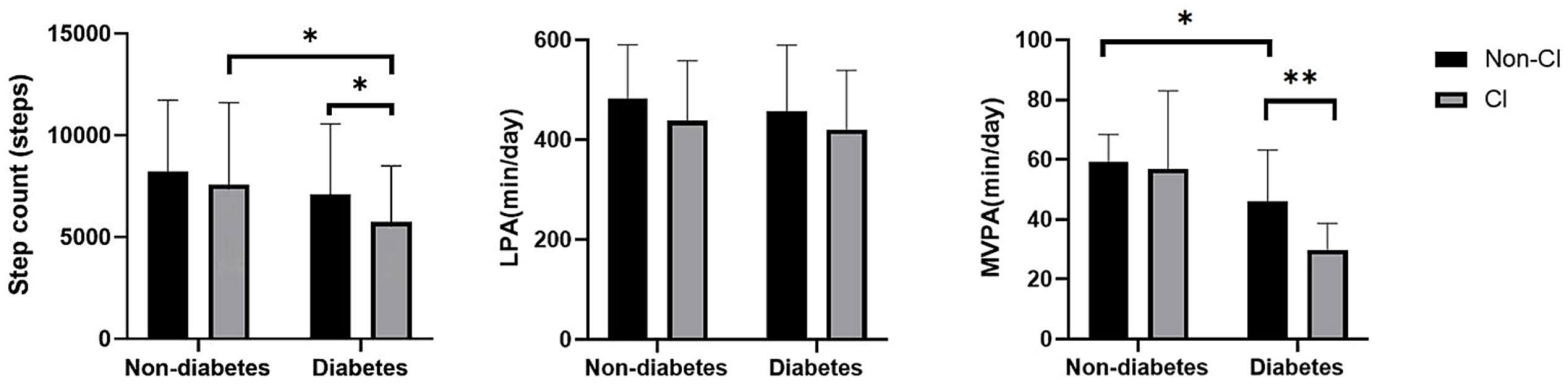
Differences in physical activity hours between groups. MVPA, moderate to vigorous physical activity level; LPA, light activity level; CI, cognitive impairment. Data are presented as mean ± SD using *t*-tests, * represents *p* < 0.05 and ** represents *p* < 0.01.

Figure 1 shows the flow of the grouping of hemodialysis participants, a total of 339 participants were involved in the analysis of this study (mean age 67.38 ± 8.07 years; 37.8% females). Among all participants, 202 (59.6%) reports diabetes and 73 (21.5%) had CI. Table 1 presents the characteristics of the study participants stratified by diabetes. Compared to non-CI, CI patients with diabetes tended to have a lower level of education and were more likely to be widowed (*p* < 0.05). As shown in Figure 2, it is worth noting that the average daily steps and MVPA duration of diabetic patients are significantly less than those of non-diabetic patients. In addition, in the diabetic group, the steps and MVPA duration of CI patients were significantly less than those in the cognitively normal group (*p* < 0.05). However, there was no statistical difference between CI group and LPA group (*p* > 0.05).

**Table 1:** Baseline information of elderly hemodialysis patients by diabetes and CI.

Characteristics	Non-Diabetes (*N* = 137)		*p* value	Diabetes (*N* = 202)		*p* value
	Non-CI (*N* = 113)	CI (*N* = 24)		Non-CI (*N* = 153)	CI (*N* = 49)	
Age(years)	67.19 ± 8.42	71.17 ± 7.07	0.033	66.36 ± 7.28	69.18 ± 9.35	0.057
Sex (%)			0.358			0.194
Male	68(60.2)	12(50.0)		103(67.3)	28(57.1)	
Female	45(39.8)	12(50.0)		50(32.7)	21(42.9)	
BMI (kg/m^2^)	22.73 ± 3.27	22.09 ± 3.01	0.381	23.61 ± 3.61 a	24.02 ± 3.71 b	0.495
Dry weight(kg)	51.26 ± 22.54	51.69 ± 19.56	0.055	54.10 ± 25.49	52.41 ± 26.07	0.329
Smoking (%)	16(14.2)	2(8.3)	0.443	36(23.5)	7(14.3)	0.169
Drinking (%)	14(12.4)	3(12.5)	0.988	17(9.9)	3(6.0)	0.431
Widowed (%)	11(9.7)	3(12.5)	0.685	14(9.2)	10(20.4)	**0.034**
Living alone (%)	17(15.0)	5(20.8)	0.483	27(17.6)	13(26.5)	0.174
Education (%)			0.074			**0.049**
Less than high school	73(64.6)	20(83.3)		85(55.6)	35(71.4)	
High school or higher education	40(35.4)	4(16.7)		68(44.4)	14(28.6)	
MMSE	27.02 ± 3.75	19.48 ± 4.98	<0.001	25.09 ± 6.96 a	19.78 ± 4.67	<0.001
CCI	3.51 ± 1.55	3.75 ± 1.59	0.500	4.76 ± 1.67 a	4.88 ± 1.84 b	0.689
Vintage (months)	58.75(30.12,118.28)	44.53(31.03,94.78)	0.184	37.86(19.18,71.24) a	46.86(19.80,70.16)	0.893
TUGT	10.10 ± 7.41	9.10 ± 4.22	0.523	10.77 ± 6.61	12.69 ± 7.67	**0.009**
Physical activity levels						
LPA (min/day)	481.64 ± 108.91	437.71 ± 120.60	0.080	458.27 ± 131.63	420.41 ± 118.74	0.074
MVPA (min/day)	59.41 ± 48.24	56.91 ± 61.76	0.827	46.14 ± 40.46 a	29.91 ± 30.50	**0.011**
Daily steps	8226.11 ± 3498.48	7582.06 ± 4072.04	0.428	7088.76 ± 3464.16 a	5741.05 ± 2754.04 b	**0.014**
Laboratory parameters						
Hemoglobin (g/L)	113.96 ± 13.75	116.04 ± 16.96	0.627	109.07 ± 13.61 a	102.65 ± 26.09 b	0.104
Albumin (g/L)	40.17 ± 3.05	39.47 ± 3.71	0.179	39.08 ± 3.84 a	38.56 ± 3.42	0.400
Phosphate (mmol/L)	1.95 ± 0.62	2.14 ± 0.72	0.061	1.81 ± 0.58	1.77 ± 0.63 b	0.737
PTH (pg./ml)	399.98 ± 348.22	437.67 ± 464.07	0.573	313.75 ± 232.43 a	347.89 ± 253.12	0.390
Calcium (mmol/L)	2.21 ± 0.23	2.12 ± 0.21	0.037	2.19 ± 0.23	2.11 ± 0.40	0.058
Kt/V	1.37 ± 0.31	1.35 ± 0.39	0.781	1.31 ± 0.32	1.34 ± 0.31	0.991
eGFR	6.63 ± 9.50	3.82 ± 2.92	0.434	4.74 ± 2.06	4.96 ± 3.26	0.573

CI, cognitive impairment; BMI, body mass index; LPA, Light physical activity; MVPA, moderate to vigorous intensity physical activity; MMSE, Mini-Mental State Examination; CCI, Charlson Comorbidity Index; PTH, parathyroid hormone, Kt/V, fractional clearance index for urea. Data are presented as mean ± SD or *n* (%). **(a)** In non-CI patients, the Diabetes group vs. the Non-Diabetes group, p < 0.05. **(b)** In CI patients, the Diabetes group vs. the Non-Diabetes group, *p* < 0.05. The bold values represents *p* < 0.05.

We used logistic regression analysis to explore the association between physical activities and cognitive impairment in hemodialysis patients (Table 2). After adjustment for potential confounders (age, sex, BMI, widowhood, education level, TUGT, and CCI), we found that each 1,000 step increase was significantly associated with a 9% reduction in the risk of cognitive impairment in hemodialysis patients (OR = 0.914, 95%CI = 0.837, 0.997), while MVPA and LPA were not significantly associated with cognitive impairment. However, in the subgroup analysis, the crude model showed a 13% reduction in the risk of prevalence of CI in the diabetes group for each 10-min increase in MVPA (OR = 0.869, 95% CI = 0.796–0.71, *p* < 0.05). In the adjusted model (age, sex, BMI, widowed, education level, TUGT and CCI), each 10-min increase in MVPA was associated with an 10% lower risk of CI prevalence in the diabetes group (OR = 0.891, 95% CI = 0.79–0.99, *p* < 0.05), suggesting that longer MVPA duration was associated with a lower odd of prevalence of CI. However, this association was absent in the non-diabetic group (OR = 1.018, 95%CI = 0.916, 1.130, *p* > 0.05).

**Table 2:** Logistic regression analysis of physical activities and CI in the non-diabetic and diabetic hemodialysis patients.

Variables	Unadjusted		Adjusted model	
	OR (95% CI)	*p* value	OR (95% CI)	*p* value
Total (*n* = 339)				
MVPA (increment per 10 min/day)	0.924(0.860,0.992)	0.029*	0.942(0.872,1.018)	0.131
LPA (increment per 30 min/day)	0.930(0.859,1.007)	0.075	0.938(0.862,1.020)	0.135
Daily steps (increment per 1000steps/day)	0.896(0.825,0.973)	0.009*	0.914(0.837,0.997)	0.043*
Non-Diabetes (*n* = 137)				
MVPA (increment per 10 min/day)	0.990(0.905,1.083)	0.826	1.018(0.916,1.130)	0.744
LPA (increment per 30 min/day)	0.899(0.796,1.014)	0.083	0.898(0.794,1.015)	0.084
Daily steps (increment per 1000steps/day)	0.949(0.833,1.080)	0.425	0.968(0.842,1.113)	0.650
Diabetes (*n* = 202)				
MVPA (increment per 10 min/day)	0.869(0.778,0.971)	0.013*	0.901(0.791,0.997)	0.046*
LPA (increment per 30 min/day)	0.933(0.864,1.007)	0.076	0.937(0.866,1.014)	0.107
Daily steps (increment per 1000steps/day)	0.874(0.784,0.975)	0.015*	0.894(0.797,1.002)	0.054

Model 1 was adjusted for age, sex and BMI. Model 2 was adjusted for age, sex, BMI, educational levels, TUGT, widowed and CCI; MVPA, moderate to vigorous intensity physical activity; BMI, body mass index; CCI, Charlson Comorbidity Index; *represents *p* < 0.05.

Then, we performed multivariate linear regression analyses of the association between physical activities and cognitive functions in non-diabetic and diabetic hemodialysis patients (Table 3). In the fully adjusted model, in the diabetic group, MVPA was positively correlated with total cognition (*β* = 0.270, 95%CI = 0.065, 0.475), temporal orientation (β = 0.053, 95%CI = 0.004, 0.103), attention and calculation (β = 0.061, 95%CI = 0.001, 0.121), and recall (β = 0.068, 95%CI = 0.021, 0.115), and daily steps was positively correlated with total cognition (β = 0.281, 95%CI = 0.052, 0.510) and orientation to time (β = 0.067, 95%CI = 0.013, 0.122), while LPA was only positively correlated with registration (β = 0.030, 95%CI = 0.002, 0.058).

**Table 3:** Multivariate linear regression analysis of the association between different intensity physical activity levels and cognitive function in non-diabetic and diabetic hemodialysis patients.

Variables	Non-diabetes				Diabetes			
	Crude		Adjusted model		Crude		Adjusted model	
	β	*p*	β	*p*	β	*p*	β	*p*
MMSE score	0.058 (−0.102, 0.219)	0.473	−0.042 (−0.213, 0.129)	0.627	0.347 (−0.102, 0.219)	**<0.001**	0.270 (0.065, 0.475)	**0.010**
MMSE sub scores								
Orientation to time	0.006 (−0.031, 0.043)	0.746	0.001 (−0.039, 0.041)	0.970	0.066 (0.020, 0.111)	**0.005**	0.053 (0.004, 0.103)	**0.035**
Orientation to place	−0.003 (−0.026, 0.021)	0.835	−0.019 (−0.045, 0.008)	0.161	0.046 (0.008, 0.084)	**0.018**	0.029 (0.072, 0.782)	0.177
Registration	0.004 (−0.012, 0.020)	0.639	−0.005 (0.023, 0.013)	0.606	0.025 (−0.003, 0.054)	0.082	0.022 (−0.010, 0.053)	0.180
Attention and calculation	0.011 (−0.044, 0.066)	0.694	−0.002 (−0.064, 0.061)	0.959	0.072 (0.013, 0.130)	**0.016**	0.061 (0.001, 0.121)	**0.045**
Recall	0.021 (−0.016, 0.059)	0.261	0.005 (−0.036, 0.045)	0.859	0.078 (0.036, 0.120)	**<0.001**	0.068 (0.021, 0.115)	**0.005**
Language	0.017 (−0.029, 0.064)	0.468	−0.022 (−0.072, 0.027)	0.374	0.058 (−0.001, 0.117)	0.054	0.037 (−0.025, 0.099)	0.234
LPA (increment per 10 min/day)								
MMSE score	0.134 (−0.120, 0.388)	0.298	0.126 (−0.134, 0.386)	0.960	0.198 (−0.010, 0.405)	0.062	0.171 (−0.018, 0.358)	0.075
MMSE sub scores								
Orientation to time	0.028 (−0.030, 0.087)	0.336	0.041 (−0.019, 0.100)	0.181	0.032 (−0.016, 0.080)	0.190	0.028 (−0.018, 0.073)	0.234
Orientation to place	−0.013 (−0.051, 0.026)	0.514	−0.025 (−0.065, 0.015)	0.224	0.035 (−0.004, 0.075)	0.081	0.031 (−0.009, 0.070)	0.127
Registration	0.011 (−0.015, 0.038)	0.401	0.005 (−0.023, 0.032)	0.746	0.031 (0.002, 0.060)	0.038	0.030 (0.002, 0.058)	**0.038**
Attention and calculation	0.078 (−0.007, 0.162)	0.072	0.098 (0.006, 0.190)	0.037	0.054 (−0.006, 0.115)	0.078	0.041 (−0.015, 0.097)	0.152
Recall	0.009 (−0.048, 0.067)	0.746	0.009 (−0.049, 0.068)	0.749	0.016 (−0.028, 0.059)	0.484	0.015 (−0.027, 0.058)	0.484
Language	0.019 (−0.055, 0.094)	0.606	−0.001 (−0.076, 0.075)	0.986	0.025 (−0.035, 0.085)	0.413	0.022 (−0.035, 0.079)	0.451
Daily steps (increment per 1000stpes/day)								
MMSE score	0.101 (−0.125, 0.327)	0.381	0.015 (−0.233, 0.253)	0.898	0.406 (0.177, 0.635)	**<0.001**	0.281 (0.052, 0.510)	**0.017**
MMSE sub scores								
Orientation to time	0.016 (−0.036, 0.068)	0.544	0.020 (−0.035, 0.076)	0.474	0.086 (0.034, 0.139)	**0.001**	0.067 (0.013, 0.122)	**0.016**
Orientation to place	−0.005 (−0.038, 0.029)	0.784	−0.022 (−0.059, 0.015)	0.234	0.064 (0.020, 0.108)	**0.004**	0.045 (−0.003, 0.092)	0.064
Registration	0.010 (−0.013, 0.033)	0.407	0.003 (−0.023, 0.028)	0.208	0.041 (0.008, 0.074)	**0.016**	0.034 (−0.001, 0.069)	0.056
Attention and calculation	0.046 (−0.013, 0.123)	0.240	0.045 (−0.042, 0.131)	0.309	0.078 (0.010, 0.146)	**0.024**	0.050 (−0.017, 0.117)	0.144
Recall	0.015 (−0.038, 0.068)	0.576	−0.003 (−0.060, 0.054)	0.916	0.060 (0.010, 0.109)	**0.018**	0.040 (−0.013, 0.093)	0.140
Language	0.016 (−0.049, 0.081)	0.629	−0.028 (−0.097, 0.041)	0.426	0.073 (0.004, 0.141)	**0.037**	0.043 (−0.026, 0.112)	0.222

Adjusted model: age(years), sex, BMI (kg/m^2^), education levels, widowed, TUGT and CCI; MVPA, moderate to vigorous intensity physical activity; LPA, light intensity physical activity; BMI, body mass index; CCI, Charlson Comorbidity Index; MMSE, Mini-Mental State Examination. The bold values represents *p* < 0.05.

## 4 Discussion

The main findings of our study show that diabetic hemodialysis patients had fewer daily steps as well as fewer physical activities, especially MVPA, compared to the non-diabetic group. Further studies found that more MVPA was significantly negatively associated with cognitive impairment in hemodialysis patients with diabetes, while no such association was found in patients without diabetes. In addition, multiple linear regression analyses showed that MVPA was positively associated with orientation to time, attention, calculation, and recall functions, daily steps was positively associated with orientation to time in diabetic hemodialysis patients.

Previous studies have shown significant differences in physical activity levels based on CI status among Chinese older adults with a mean age of 68.7 years (26). Adverse health outcomes such as diabetes are strongly associated with physical activity and dietary patterns, and are also risk factors for CKD (13, 27). Therefore, in this study we compared physical activity levels of different intensities in the hemodialysis population under diabetic and CI subgroups, and explored the relationship between physical activity levels and CI among diabetic and non-diabetic hemodialysis patients. Notably, we found that MVPA duration was worse in diabetic CI patients compared to the diabetic non-CI group (Figure 2; *p* < 0.05) but the difference was not significant in the non-diabetic group. Given the significant muscle mass loss present in hemodialysis patients, the accompanying muscle dysfunction in turn limits their mobility. Therefore, physical activity levels measured using accelerometers showed lower levels in the group of hemodialysis patients compared to healthy individuals of comparable age (6, 28). Studies have shown that lower limb muscle mitochondrial oxidative capacity is reduced by 25% in patients with chronic kidney disease, which is a significant predictor of exercise performance, and that a history of comorbid diabetes likewise leads to reduced mitochondrial function (29). Thus, altered metabolic transcriptional networks and defective mitochondrial function may be one of the important mechanisms by which diabetes impairs physical function in the progression of chronic kidney disease. Although a study on the comparison of physical function between diabetic groups showed that hemodialysis patients in the diabetic group had significantly lower physical function than those in the non-diabetic group (30), there are no studies that have demonstrated that objectively measured levels of physical activity are significantly reduced in the presence of coexisting diabetes and CI.

In addition, the results of our study showed a higher prevalence of CI in diabetic hemodialysis patients (24.3%). This finding has similarities with the AGES-Reykjavik study (31), which showed that older adults with diabetes performed more poorly on cognitive tests compared to those without diabetes. There are several mechanisms that contribute to this result. First, the accumulation of advanced glycosylation end products is one of the key factors leading to vascular endothelial dysfunction, which in turn triggers multiple risk factors including oxidative stress, inflammation, vascular calcification, and insulin-like growth factor-1, which play an important role in the progression of cognitive impairment (17). Secondly, neurodegeneration has also been proposed to explain the association between diabetes and cognitive impairment. Insulin receptors are heavily distributed in the hippocampus, internal olfactory cortex, and frontal lobes, and insulin may affect cognitive function by modulating cortical activity and brain metabolism, as well as controlling the production of the neurotransmitter acetylcholine (32). Thus, diabetes may be an important risk factor for CI progression in patients with end-stage renal disease undergoing hemodialysis. However, it has also been suggested that diabetes does not independently predict an increased risk of cognitive impairment in patients with MHD (18), and the inconsistent results may be due to differences in the tools used to screen for cognitive impairment and differences in the age range of the population.

Previous studies have shown a significant negative association between self-reported physical activity levels and CI in maintenance hemodialysis patients (12), but self-reported physical activity is susceptible to recall bias and do not include a classification of PA intensity. Our study, which used an objective measure of physical activity by accelerometer, did not find a significant association between MVPA and cognitive impairment in hemodialysis patients, which is inconsistent with previous studies (26) and may be related to the characteristics of the study population, the overestimation of physical activity duration caused by the wearing of the accelerometer on the wrist, different criteria for assessing cognitive function, and the stronger influence of factors related to renal disease, such as proteinuria, etc. (33). Several studies have shown that varying degrees of decreased kidney function (defined by glomerular filtration rate) are associated with cognitive impairment (34, 35). Specifically, for every 10 mL/min/1.73m^2^ increase in eGFR, the risk of cognitive decline was reduced by 4.8% (36), which may be due to brain endothelial dysfunction caused by inflammation and toxin accumulation, leading to cognitive impairment in patients with chronic kidney disease (CKD). However, no significant difference was found between eGFR after grouping by CI status in our study, which may be related to the low level of renal function and low eGFR dispersion in hemodialysis patients in this study.

However, after grouping our patients based on the presence or absence of diabetes, we observed a significant negative correlation between MVPA duration and CI only among hemodialysis patients in the diabetes group. Recent studies have shown that performing longer periods of physical activity shows a significant correlation with a reduced risk of developing diabetes (15) and slowing the progression of diabetes to CKD (14). In addition, it was concluded that diabetes is associated with an increased risk of cognitive impairment in hemodialysis patients (16). A study has found that impaired physical performance, such as slower gait speed, was an early marker of cognitive dysfunction (37). DM may affect muscle function through several mechanisms. Peripheral insulin resistance decreases the glucose uptake to muscle and reduces muscle tissue anabolic rates (38). Moreover, a study has shown that hyperglycemia is associated with weakness as well as mobility limitations, which may be mediated by loss of muscle (39). This is also consistent with previous findings supporting a role of the diabetes in the association between muscle function and cognitive decline (17). Recent longitudinal studies in middle-aged and older diabetic populations have shown similar results, suggesting that physical activity may halt some of the potential decline in cognitive function over a 2-year period (5), while another study indicated that diabetic patients with lower levels of physical activity were at higher risk for dementia compared with nondiabetic patients with higher levels of physical activity (19). Possible mechanisms linking physical activity and cognitive function in diabetic patients: on the one hand, it may be due to the neurotoxicity of hyperinsulinemia, where increased insulin sensitivity after physical activity may favors neurogenesis and thus cognitive function (40); on the other hand, the energy expenditure associated with physical activity may reduce cognitive deficits resulting from insulin resistance through vascular mechanisms (41). In addition, we did not find a significant association between LPA and CI in either the diabetic or non-diabetic group, which is similar to the study by Katherine et al. (42) which showed that LPA was not associated with overall cognitive function in older adults but may be more sensitive to subtle changes in cerebral vascular function (i.e., cerebral blood flow, CBF). These findings further clarify the susceptibility factors for CI, and moderate-to-vigorous physical activity interventions in a more accurate population may help in the early prevention and control of the progression of CI.

Additionally, after adjusting for potential confounders, we found that in the diabetes group, MVPA was positively associated not only with overall cognitive functioning, but also with a number of specific functions, such as temporal orientation, attention, and computation and recall. According to the most recent study, longer periods of MVPA were significantly associated with improvements in memory and executive function in cognitively healthy older adults (42). Additionally, higher MVPA was found to be positively associated with increased attentional processing speed (43) and improved situational memory (5) in people with diabetes, findings similar to those in this study. The following clinically relevant associations explain our results: cognitive functioning is associated with the dorsolateral frontal cortex and the hippocampus, which collectively affect an individual’s executive functioning, attentional and computational abilities, and recall (17). In addition, exercise is associated with an increase in brain volume in specific areas of the brain related to executive function and memory (e.g., hippocampal volume, etc.) (44), and promotes higher levels of brain-derived neurotrophic factor (45), which improves functioning in cognitive domains. Currently, while many studies have shown a relationship between physical activity and cognitive function, some inconsistent results remain. Future research should focus on cognitive changes in physically weak populations, and more well-designed cohort studies are needed to validate the relationship between different levels of intense physical activity and different cognitive functions. Overall, the results of our study shed light on how to manage activity levels and intervene in CI in hemodialysis patients, especially those with diabetes.

This study presents several strengths. First, the study used a triaxial accelerometer to assess different intensifies of physical activity (including LPA and MVPA), and the accelerometer can obtain objective data in a non-laboratory setting. Secondly, this study is the first multi-center study to analyze the relationship between objectively measured physical activity levels of different intensity and cognitive impairment and multiple cognitive functions in hemodialysis patients in different diabetic states. However, there are some limitations. First, this study was limited to recruiting participants from a single city, resulting in a limited sample size and limitations in regional representation. In the future, we plan to further expand the sample size, extend the time span of the study, and expand the scope of the study to a wider range of age groups, including the super-elderly or young hemodialysis population, in order to obtain more comprehensive study results. Second, the study did not take into account other factors that may contribute to cognitive impairment, such as urinary albumin, etc. In the future, we will do further research to explore the relevant factors. Third, while wristband accelerometers improve participants’ comfort, they may overestimate physical activity metrics, such as MVPA and step count, and provide only varying intensities of PA without exploring the type and complexity of activity. Finally, this is a cross-sectional study and further longitudinal studies are needed to explore the emerging risk of CI in the diabetic hemodialysis population. In addition, we will further discuss and analyze other groupings, such as hypertension, that may produce similar results to our current study in future studies.

## 5 Conclusion

In conclusion, in this study we found a significant negative association between moderate to vigorous physical activity duration and cognitive impairment in diabetic hemodialysis patients, however, this association was not found in the non-diabetic group. In addition, we analyzed the associations between physical activity levels and specific cognitive functions to further understand the mechanisms of the interactions. Further confirmation of the causal relationship between physical activity levels and cognitive impairment in diabetic hemodialysis patients is needed in the future. This study is important for early differential diagnosis of CI and improvement of cognitive status in hemodialysis patients.

## Data Availability

The original contributions presented in the study are included in the article/supplementary material, further inquiries can be directed to the corresponding author.
